# Carbonic Anhydrase Inhibitors as Novel Antibacterials in the Era of Antibiotic Resistance: Where Are We Now?

**DOI:** 10.3390/antibiotics12010142

**Published:** 2023-01-10

**Authors:** Alessio Nocentini, Clemente Capasso, Claudiu T. Supuran

**Affiliations:** 1 NEUROFARBA Department, Section of Pharmaceutical and Nutraceutical Sciences, University of Florence, 50019 Firenze, Italy; 2 Department of Biology, Agriculture and Food Sciences, Institute of Biosciences and Bioresources, CNR, 80131 Napoli, Italy

**Keywords:** carbonic anhydrase, sulfonamides, inhibitors, antibiotic resistance, bacteria

## Abstract

Resistance to antibiotic treatment developed by bacteria in humans and animals occurs when the microorganisms resist treatment with clinically approved antibiotics. Actions must be implemented to stop the further development of antibiotic resistance and the subsequent emergence of superbugs. Medication repurposing/repositioning is one strategy that can help find new antibiotics, as it speeds up drug development phases. Among them, the Zn^2+^ ion binders, such as sulfonamides and their bioisosteres, are considered the most promising compounds to obtain novel antibacterials, thus avoiding antibiotic resistance. Sulfonamides and their bioisosteres have drug-like properties well-known for decades and are suitable lead compounds for developing new pharmacological agent families for inhibiting carbonic anhydrases (CAs). CAs are a superfamily of metalloenzymes catalyzing the reversible reaction of CO_2_ hydration to HCO_3_^−^ and H^+^, being present in most bacteria in multiple genetic families (α-, β-, γ- and ι-classes). These enzymes, acting as CO_2_ transducers, are promising drug targets because their activity influences microbe proliferation, biosynthetic pathways, and pathogen persistence in the host. In their natural or slightly modified scaffolds, sulfonamides/sulfamates/sulamides inhibit CAs in vitro and in vivo, in mouse models infected with antibiotic-resistant strains, confirming thus their role in contrasting bacterial antibiotic resistance.

## 1. Introduction

**Figure 1:**
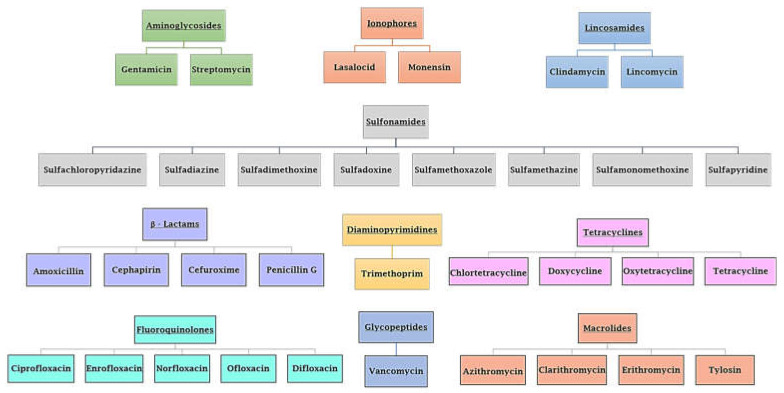
Various types of FDA-approved antibiotics. Class and antibiotic name are reported.

Antibiotic resistance is a worldwide emergency that kills more people than HIV/AIDS and malaria combined [1]. It kills around 30,000 people in Europe each year, with Italy accounting for one-third of them [2,3]. In addition, antibiotic-resistant illnesses have a significant effect on public health services [4]. Antibiotic resistance is the ability of bacteria to counteract the action of one or more of the commune FDA-approved antibiotics (Figure 1) [4,5]. The antibiotics reported in Figure 1 act as suppressors of cell wall synthesis, inhibitors of proteins or nucleic acids synthesis, membrane destroyers, antimetabolites, and competitive antagonists of substrates used in biosynthetic reactions [6]. It is essential to understand that humans and other animals do not acquire antibiotic resistance; instead, this phenomenon is developed by bacteria harbored in humans and animals [3]. When placed under selective pressure due to antibiotics, bacteria that have acquired a greater capacity for resistance (by DNA mutation or DNA genetic transfer) will have a greater chance of surviving and instead will occupy the environment vacated by bacteria that have been eliminated by therapy [7].

Thus, to treat infections caused by those resistant bacteria, one strategy is to administer other antibiotics to which they are sensitive. However, they might also acquire resistance to the new class of antibiotics (multi-resistant organisms), and so switching to a new type of antibiotic is required until we arrive at bacteria resistant to all antibiotics (pan-resistant microorganisms) [8]. The antibiotic resistance phenomenon is associated with the misuse and overprescribing of these drugs, as well as the inappropriate administration of antibiotics to companion animals and animals in the agriculture industry [9,10]. In livestock farms or aquacultures all over the world, antibiotics are routinely used not only to treat diseases, as is the case in human medicine but also to prevent diseases and as promoters of animal growth [11]. Furthermore, the rising discharge of antibiotics into waterways and soils poses a risk to all microorganisms in these habitats [11]. Thus, bacteria can become resistant and infect people who came in touch with the polluted environment, animals, or meat. Therefore, policies must be put in place to combat both the spread and future development of antibiotic resistance, as well as the oncoming wave of superbugs [12].

How do we intervene to stop antibiotic resistance? There is no shortage of strategies for dealing with the antibiotic resistance [13,14,15,16]. Among them, one may consider: (i) community- and healthcare-based approaches for infection control and prevention; (ii) vaccine preparation, which may have a good chance of preventing bacterial illnesses (up to date, only for *Streptococcus pneumoniae*, one of the six most hazardous antibiotic-resistant bacteria, exists such a vaccine); (iii) reduction of the use of antibiotics in non-human infection-treatment contexts, such as livestock farms; (iv) appropriate antibiotic use, as well as stopping their use for the management of viral infections; (v) maintenance of investments to make second-line antibiotics available, and the development of novel antibiotics and especially those with novel modes of action less susceptible to the onset of resistance, which can save lives [13,14,15,16]. This last strategy is fundamental, but new drug development takes a long time; most candidate compounds were in the research and development pipelines for over a decade before they made it to market [17]. Moreover, the most critical points are that the majority of the new antibiotics in development are variants of preexisting antibiotic classes, do not have a novel mechanism of action, and only a small number of them are expected to be effective against the **ESKAPE** pathogens (***E****nterococcus faecium*, ***S****taphylococcus aureus*, ***K****lebsiella pneumoniae*, ***A****cinetobacter baumanii*, ***P****seudomonas aeruginosa*, and ***E****nterobacter*). Finally, the future of antibiotics is shaky because of questions about their effectiveness and safety [18,19].

In this context, the drug repurposing or drug repositioning strategy offers the potential to “fast-track” the identification of novel antibiotics, since it speeds up the drug research process and reduces the time to market [20]. FDA-approved medications are considered the “existing drugs,” and their repurposing reduces clinical development risk, making them attractive candidates [20].

## 2. A Superfamily of CO_2_ Transducer Biomolecules: The Carbonic Anhydrase

### 2.1. Bacterial Carbonic Anhydrase

Carbon dioxide (CO_2_) is a gas released into the atmosphere due to cellular respiration and oxidative metabolism, and all living creatures are responsible for its production [21]. In most cases, the process of transporting this waste gas out of cells is carried out by passive diffusion [22]. In many cases, the CO_2_ channels, controlled by CO_2_ levels inside the cell, make this transfer easier. However, CO_2_ is not merely a waste product; it can also trigger various cellular signaling pathways to increase bacteria virulence and pathogenicity [23]. Bacteria can adapt to their environment by sensing and responding to CO_2_, enhancing thus their chances of survival. This may be crucial because bacteria have to adapt to the relatively low CO_2_ levels of the outer atmosphere for the higher CO_2_ levels found inside most multicellular host organisms [23]. Bacteria, for example, may upregulate virulence factors at host physiologic CO_2_ levels rather than ambient CO_2_ levels to aid colonization or infection. Several such examples are *Vibrio cholerae*, which causes cholera, and produces enterotoxin as carbon dioxide levels rise, whereas bicarbonate produced by the CO_2_ hydration is the first positive effector of the primary *V. cholerae* virulence gene transcription activator (ToxT), responsible for the cholera virulence cascade [24]. *Pseudomonas aeruginosa*, which can lead to infections in the blood, lungs (pneumonia), or other regions of the body following surgery, lives in vastly varying CO_2_ settings depending on whether or not it is colonizing a host [25].

Biomolecules in microbes related to CO_2_-sensitive pathways or acting as a CO_2_ transducer have been proposed as appealing targets for medicines, since they control cell development and the subsequent synthesis of chemicals, enhancing the pathogen persistence in the host [26,27]. In this context, a crucial role is played by a superfamily of molecules known as carbonic anhydrases (CAs, EC 4.2.1.1). CAs can be thought as molecules that, rather than instantly detecting a change in CO_2_, serve as CO_2_ transducers, adjusting its levels [23,28]. With their activity, the CAs encoded by the bacterial genome of pathogenic and non-pathogenic bacteria provide the indispensable CO_2_ and HCO_3_^−^/protons to microbial biosynthetic pathways, catalyzing the reversible reaction of CO_2_ hydration to HCO_3_^−^ and H^+^ (CO_2_ + H_2_O ⇋ HCO_3_^−^+ H^+^) [28]. Here, we stress the fact that the non-catalytic CO_2_ hydration/dehydration reaction is too slow at physiological pH values to fulfill the organism’s metabolic demands (k_cat (hydration)_ = 0.15 s^−1^; k_cat (dehydration)_ = 50.0 s^−1^) [29].

**Figure 2:**
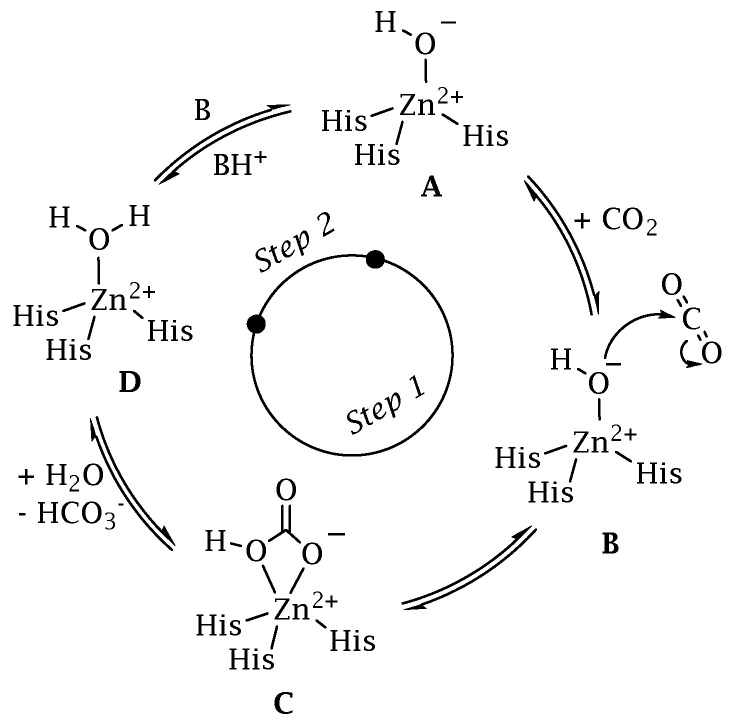
CA catalytic mechanism schematically represented for a α-class isoform.

**Figure 3:**
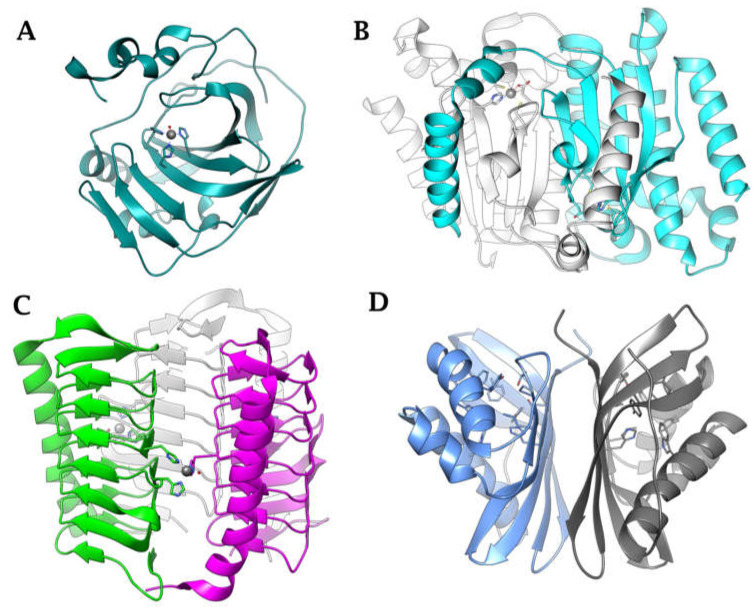
Ribbon view of (**A**) α-CA from *Neisseria gonorrhoeae* (PDB 1KOQ), (**B**) β-CA from *Escherichia coli* (PDB 1I6O), (**C**) γ-CA from *Burkholderia pseudomallei* (PDB 7ZW9), (**D**) ι-CA from *B. territorii* built by homology using PDB 3H51 as a template.

The classification system for CAs uses the Greek letters to represent the eight distinct families (or classes): α, β, γ, δ, ζ, η, θ, and ι [29,30,31,32,33]. The eight distinct CA-classes descend from the same ancestor, yet exhibit significant evolutionary diversity. The representative amino acid sequences of each CA-class show low sequence similarity, characteristic folds, and structures compared to the polypeptide chain of other CAs belonging to a different class [34,35,36,37,38]. In contrast, the mechanism involved in the reversible hydration of CO_2_ is strictly conserved across all CA-classes, illustrating the CA superfamily’s convergent evolution [34,35,36,37,38]. CAs are generally metalloenzymes with catalytic sites that contain a metal ion cofactor required for catalysis (Figure 2). The ion cofactor in many CAs is Zn^2+^, which is coordinated by three amino acid residues from the protein backbone [29,30,39]. The fourth metal ion ligand is a water molecule/hydroxide ion that acts as the nucleophile in the enzyme’s catalytic cycle. Metal ions other than Zn^2+^, such as Co^2+,^ Cd^2+^, Fe^2+^, and Mn^2+^, can be coordinated by several CA-classes [40,41,42,43,44,45,46,47]. Recently, it has been demonstrated that the newly discovered CA-class, the ι-CA, shows catalytic activity without the need for metal ions, as shown from the X-ray crystal structure of *Anabaena* sp. [48]. The CA-classes differ in the amino acid residues involved in metal coordination [49,50,51,52]. For example, the ion metal is coordinated by three His residues in the α, β, and γ-CAs and presumably θ-classes [42,45,53,54]; one His and two Cys residues in the β- and ζ-CAs [41]; and two His and one Gln residue in the η-class. α-CAs usually act as monomers or dimers; β-CAs only behave as dimers, tetramers, or octamers. To perform their catalytic activity, the γ-CAs must be trimers [55]. A tandemly repeated hexapeptide characterizes γ-CA monomers and is required for the left-hand fold of trimeric-helix structures. The X-ray structure of the θ-CAs was remarkably similar to that of some β-CAs [42,45,53,54]. The crystal structure of ζ-CA showed three slightly different active sites on the same polypeptide chain. Regarding the structural organization of δ- and η-CAs, no data are currently available, and a homology modelling of the η-CA was built [56]. Interestingly, only the α, η, θ, and ι -CAs have been shown to catalyze the esters/thioesters hydrolysis, while the other CA families lacked any detectable esterase activity [29,30,39,57,58,59]. Presently, four CA-classes (α, β, γ, and ι) have been demonstrated to exist in bacteria, and their distribution is noteworthy [34,35,36,37,38]. In many cases, the bacterial genome encodes for the three CA-classes (α, β, and γ) and rarely for the ι- class. However, it is common to find bacteria whose genomes encodes just one or two CAs, and very rarely none [27,29,33,49]. Moreover, the structural variations between bacterial and human α-CAs allow for the synthesis of inhibitors that target the bacterial enzyme but not the mammalian ones. Again, in some cases, the pathogenic bacterial genome encodes for CA-classes, which are absent in mammals, whose genome encodes only for α-CA, increasing the likelihood of success in treating the bacterial illness with compounds that act only on the bacterial CAs. Interestingly, for each CA-class has been obtained the X-ray crystallographic structures (Figure 3). It has been observed that α-CAs reside in the periplasmic region of bacteria cells and prevent CO_2_ loss from bacteria by converting CO_2_ into bicarbonate, which is then transported inside the cytoplasm by bicarbonate transporters [29,30,31,33]. On the other hand, cytoplasmic β- and γ -CA classes are responsible for carrying out intracellular functions such as maintaining CO_2_ and HCO_3_^−^ equilibrium and regulating pH. Recently, β, γ, and ι-CAs with signal peptide at N-terminus have been found, attributing them a putative periplasmic localization and a physiological role similar to those mentioned above for the α-CAs [31,32,39,57,58,59].

### 2.2. CAs Help Bacteria to Survive

In the literature, many shreds of evidence support the opinion that the activity of CAs is connected to the survival of microbes because these enzymes are essential for supporting numerous physiological functions involving dissolved inorganic carbon, such as transport and supply of CO_2_ or HCO_3_^−^, pH homeostasis, secretion of electrolytes/toxins, and biosynthetic processes [29,33,60]. For example, it has been proven in vivo that bacterial growth at ambient CO_2_ concentrations is dependent on CA activity in bacteria such as *Ralstonia eutropha* (Gram-negative bacterium found in soil and water) and *Escherichia coli* (Gram-negative bacterium) [61,62,63]. In *E. coli*, the two β-CAs (CynT and CynT2) generate HCO_3_^−^ to prevent bicarbonate depletion from cyanate breakdown and bacterial expansion at atmospheric CO_2_ concentrations, respectively [62,63]. More intriguing is the in vivo evidence that CAs have a role in the proliferation of harmful bacteria such as *Mycobacterium tuberculosis* [64,65,66,67,68], *Helicobacter pylori* [69,70,71], *Vibrio cholerae* [72], *Brucella suis* [65,66,67,68], *Salmonella enterica* [73,74,75], and *Pseudomonas aeruginosa* [76]. CAs encoded by the genome of *H. pylori*, a Gram-negative, microaerophilic bacteria that colonizes the human stomach, are essential for the pathogen’s acid acclimation and, consequently, survival in the severe environment typical of this organ, with pH values as low as 1.5–2.0 [69,70,71]. In addition to CAs, urease is the other enzymatic system used by the microbe for growing in this extreme environment. Under acidic conditions, urea goes into the cytoplasm through the urea channel. In the bacterial cytoplasm, 2NH_3_ and CO_2_ are produced by the hydrolysis of urea [69,70,71]. The resulting CO_2_ is then hydrated by β-CA, while the periplasmic α-CA hydrates the CO_2_ diffused in the periplasm. The produced ions (H^+^) by the CA-catalyzed reaction are used to form NH_4_^+^ by reacting with NH_3_^+^ in the periplasm and cytoplasm, which neutralizes the entering acid in the above environments [69,70,71]. In the case of the pathogenic bacterium *Vibrio cholerae*, a Gram-negative bacterium already mentioned above, CAs are involved in the production of sodium bicarbonate, which stimulates the development of cholera toxin [24]. It has been proposed that *V. cholerae* employs CAs to colonize the host [72]. Again, the brucellosis causal agent, *Brucella suis*, a non-motile Gram-negative coccobacillus, and *Mycobacterium tuberculosis*, a pathogenic bacterium that causes tuberculosis, were demonstrated to require functional CAs to proliferate [64,65,66,67,68]. Furthermore, through in vivo gene expression investigations on the bacterium *Salmonella enterica*, the MIG5 gene, which encodes for a CA that is significantly expressed during bacterial infection, has been found [73,74,75]. The deletion of the gene encoding this CA (psCA1) in the *Pseudomonas aeruginosa* reduced pathogenicity by decreasing calcium salt depositions [77].

### 2.3. Carbonic Anhydrase Sulfonamide Inhibitors

Because the many biochemical processes mentioned above involve the activity of bacterial CAs, their inhibition may reduce the pathogen’s survival and fitness. The good news is that CA inhibition suppresses bacterial growth differently from those demonstrated by traditional antibiotics, toward which the bacteria have developed or are developing antibiotic resistance. As reported in the scientific literature, many unique chemical classes of CA inhibitors (CAIs) exist [60]. The CAIs are classified into four distinct types based on how the inhibitors bind and inhibit the CA metalloenzymes. Four types of inhibitor-enzyme binding are currently known, based on whether the binding involves the catalytic metal ion or the metal coordinated-water molecule or how the active site is obstructed [78]. Therefore, there are metal ion binders (anion, sulfonamides and their bioisosteres, dithiocarbamates, xanthates, and so on); chemicals that bind to the zinc-coordinated water molecule/hydroxide ion (phenols, polyamines, thioxocoumarins, sulfocumarins); compounds that obstruct the active site entrance (coumarins and related isosteres); and compounds that bind out of the active site (carboxylate) [78].

**Figure 4:**
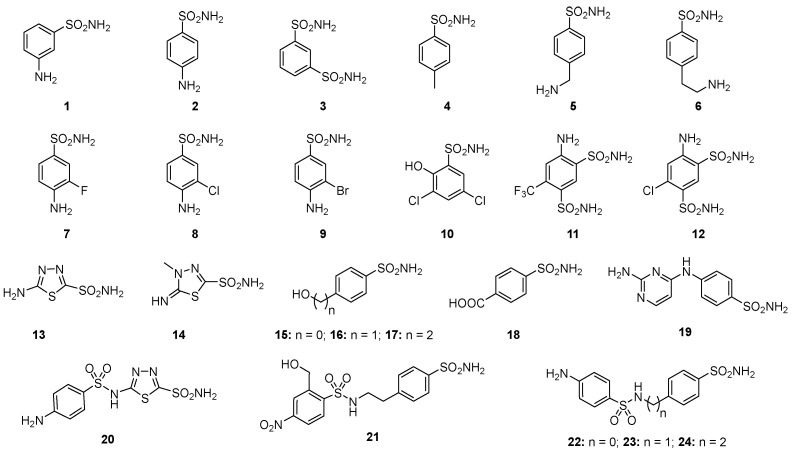
Structure of commercially available sulfonamide derivatives **1**–**24**.

**Figure 5:**
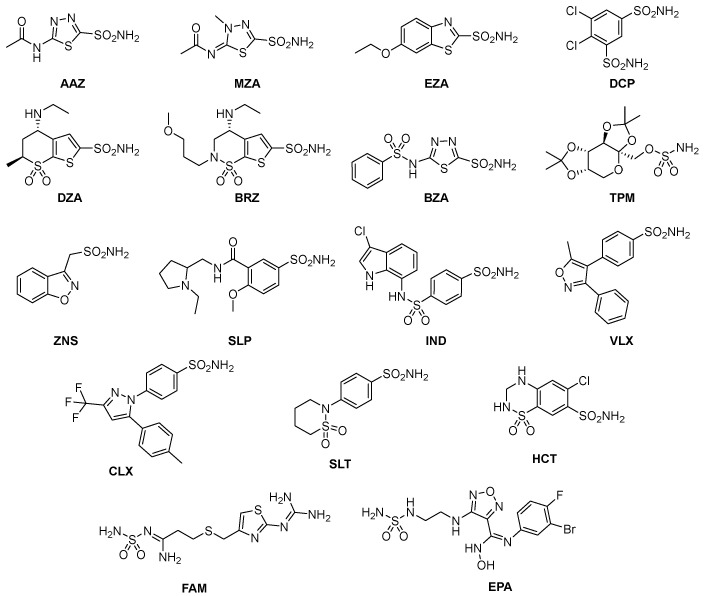
Structure of clinically used sulfonamide/sulfamate/sulfamide derivatives **AAZ**-**HCT**.

**Figure 6:**
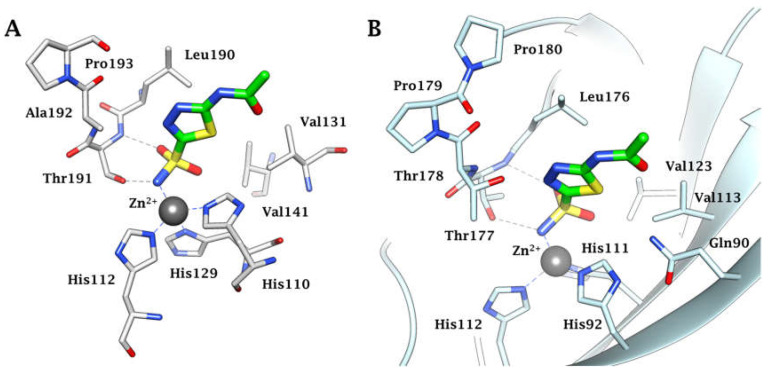
(**A**) Active site view of the α-CA of *Helicobacter pylori* in adduct with **AAZ** (4YGF). (**B**) Active site ribbon view of the α-CA of *N. gonorrhoeae* in adduct with **AAZ** (8DYQ). H-bonds are represented as black dashed lines.

Among these types, the Zn^2+^ ion binders, in particular, the sulfonamides and their bioisosteres, are considered the most promising compounds for the realization of novel antibacterials, avoiding antibiotic resistance [78,79,80,81]. Here, we emphasize that the sulfonamides/sulfamates/sulfamides able to inhibit the CA specifically are nonantibiotic inhibitors characterized by a primary sulfonamide moiety, having the following chemical formula: R-X-SO_2_-NH_2_, where R can be an aromatic, heterocyclic, aliphatic, or sugar scaffold, X = nothing, O or NH. Thus, sulfanilamide led to the discovery of the sulfa drugs and benzenesulfonamide CAIs of the type. They constitute an important class of drugs since they have drug-like properties well-known for decades and are suitable lead compounds for developing new pharmacological agent families for inhibiting CAs. Among them are the commercial derivatives **1**–**24** (Figure 4) and the clinically used agent **AAZ**-**EPA** (Figure 5). The series **AAZ**-**EPA** include acetazolamide (**AAZ**), methazolamide (**MZA**), ethoxzolamide (**EZA**), and dichlorophenamide (**DCP**) are classical, systemically working antiglaucoma CAIs. Dorzolamide (**DZA**) and brinzolamide (**BRZ**) are topically acting antiglaucoma agents. Benzolamide (**BZA**) is an orphan drug belonging to this class of pharmacological agents. Zonisamide (**ZNS**), sulthiame (**SLT**), and sulfamic acid ester topiramate (**TPM**) are widely used antiepileptic drugs. Sulpiride (**SLP**) and indisulam (**IND**) were also shown by our group to belong to this class of pharmacological agents, together with the COX2 selective inhibitors celecoxib (**CLX**) and valdecoxib (**VLX**). Saccharin (**SAC**) and the diuretic hydrochlorothiazide (**HCT**) are also known to act as CAIs. Famotidine (**FAM**) and epacadostat (**EPA**) are CAI sulfamide drugs clinically used respectively as a histamine H2 receptor antagonist and a selective indoleamine-2,3-dioxygenase 1 inhibitor. These inhibitors bind Zn (II) in a tetrahedral geometry, forming an extended network of hydrogen bonds with the enzyme amino acid residues, whereas the aromatic/heterocyclic portions of the inhibitor interact with the hydrophilic and hydrophobic residues found in the enzyme catalytic cavity, according to enzyme-inhibitor X-ray crystallographic data (Figure 6) [60].

## 3. Where Are We NOW with the Inhibition of the Bacterial CAs

In the last decade, several in vitro experiments were conducted employing the CAIs outlined in the preceding paragraph to inhibit the four bacterial CA classes (α, β, γ, and ι). Many of these studies were focused on bacterial CAs derived from pathogenic bacteria, such as *Mycobacterium tuberculosis*, *Vibrio cholerae, Francisella tularensis, Burkholderia pseudomallei*, *Porphyromonas gingivalis*, *Legionella pneumophila*, *Clostridium perfringens*, *Mammaliccosu sciuri*, etc. [36,82,83,84,85]. Most sulfonamide CAIs exert potent inhibition on most recombinant CAs belonging to the bacteria mentioned above [34,86,87,88,89,90]. The most interesting aspect was that, some of these CAIs, including acetazolamide and methazolamide, significantly limit the growth of bacteria in cell cultures [91]. In this context, some experimental shreds of evidence prove that the inhibition of bacteria CAs can be potentially used to combat the resistance of many pathogens to the existing antimicrobial drugs.

For example, ethoxzolamide (**EZA**), an authorized diuretic and carbonic anhydrase inhibitor, kills *Helicobacter pylori* in vitro, suggesting it could be turned into an anti-*H. pylori* medication [92].

The influence of the selective CA inhibitor **AAZ** on the bacterial lifecycle was tested by analyzing the growth of *E. coli* and its consumption of glucose, added as the only carbon source to the bacterial culture media [93]. Carbon sources are required for biosynthetic activities and metabolism is directly correlated with the rate at which carbon sources are used. The FDA-approved carbonic anhydrase **AAZ** was able to interfere with *E. coli* growth and glucose uptake at 31.2 μg/mL. Intriguingly, **AAZ** resulted in a good inhibitor of the two recombinant *E. coli* CAs, β-CA (CynT2) and γ-CA (EcoCAγ), with a K_I_ of 227 and 248 nM, respectively [94,95]. AAZ prevents sugar consumption due to its inhibitory action on bacterial CAs, which are directly engaged in providing CO_2_/HCO_3_^−^ required for the bacterial metabolic need [93].

It has been reproposed the FDA-approved carbonic anhydrase drug **AAZ** could be used to design potent antienterococcal agents. The authors, modifying the **AAZ** scaffold, arrived at two leads possessing improved potency against clinical vancomycin-resistant enterococci (VRE) strains [96]. The classical **AAZ** showed a MIC of 2 μg/mL, while the two leads had a MIC = 0.007 μg/mL and 1 μg/mL, respectively. **DZA**, another classical CAIs, resulted in MIC values of 1–8 μg/mL against a panel of clinical VRE isolates [97]. Based on the results of homology modeling and molecular dynamics simulations, the authors demonstrated that the α and γ CAs encoded by the Vancomycin-Resistant Enterococcus are the intracellular targets of the compounds [96,98]. In addition, **AAZ** fared better than linezolid (a standard drug for VRE infections) when tested in two in vivo VRE mouse models –murine colonization–reduction and VRE septicemia [99].

Finally, **AAZ** inhibited the growth of the Gram-negative bacterium *Neisseria gonorrhoeae* both in the in vitro as well as in vivo mouse model of a gonococcal genital tract infection [100,101]. Recently, Portela et al. [102] demonstrated that sulfonamide pretreatment has a positive outcome on the strength of dentin and the prevention of *Streptococcus mutans* colonization in teeth treated with two potent bacterial CA sulfonamide inhibitors [102].

It is also interesting to note that over the last few years, there has been a significant focus on developing non-classical CAIs for treating multiple diseases due to the prevalence of sulfonamide allergies among the general population as well as their use as potential new antibacterials [103]. The classes of non-classical inhibitors that show strong potential as lead compounds for isoform-specific drug design include phenols, polyamines, carboxylic acids, and coumarins and their derivatives. These compounds can anchor to the zinc-bound water/hydroxide ion or bind outside the active site to block substrate entry, exhibiting atypical binding mechanisms of the classical sulfonamide CAIs [103].

## 4. Conclusions

The well-known zinc-binding groups (ZBGs), such as the primary sulfonamide (-SO_2_NH_2_), primary sulfamate (-OSO_2_NH_2_), and sulfamide (-NHSO_2_NH_2_), which are present in the structure of several clinically-approved drugs and an increasing number of investigational medicines, have been proven to affect the inhibition of the CAs encoded by pathogenic bacteria [78,79,80,81]. Intriguingly, various types of these nonantibiotic sulfonamides, many of which offer pharmacologic applications as antiglaucoma, antiobesity, antitumor, or diuretic, resulted in potent inhibitors (k_I_ in the nanomolar range) of such CAs. These findings prompt scientists to consider the CAIs as a new approach to fight antibiotic resistance developed by bacteria versus the common FDA-approved antibiotics. Unlike common antibiotics, the CAIs impair the growth of pathogenic bacteria through a novel mechanism of action: perturbating/depleting the intracellular levels of CO_2_ and HCO_3_^−^, which are necessary to microbial biosynthetic pathways. In addition, the antibacterial growth due to the CA inhibition is strongly supported by the fact that the slow non-catalytic CO_2_ hydration/dehydration reaction is unable to restore these levels. These attractive facts led to the writing of fascinating manuscripts that discuss the repurposing of drugs such as ethoxzolamide (**EZA**), acetazolamide (**AZA**), and dorzolamide (**DZA**) as treatments for interfering with the life cycle of *E. coli*, *Helicobacter pylori, Neisseria gonorrhoeae,* and vancomycin-resistant enterococci.

In conclusion, the licensed sulfonamides/sulfamates/sulfamides acting as CAIs exhibit antibacterial properties in their native or slightly modified scaffolds [97], hypothesizing that the CAIs can be potentially employed either by themselves or in conjunction with an antibiotic or even as “antibiotic adjuvants” to increase the effectiveness of certain antibiotics.

## Data Availability

Not applicable.
